# Metabolic Syndrome and Pancreatic Cancer: Linking Insulin Resistance, Inflammation, and Carcinogenesis

**DOI:** 10.3390/diagnostics16162616

**Published:** 2026-08-18

**Authors:** Rahela Borbei, Vlad Dumitru Brata, Ioana Dobrotă, Ligia-Maria Ceteraș, Mihai Clim, Teodora-Gabriela Alexescu, Mircea-Vasile Milaciu, Mirela-Georgiana Perne, Cezara-Andreea Gerdanovics, Lucia Maria Procopciuc, Angela Cozma, Olga-Hilda Orășan

**Affiliations:** 1 Department of Internal Medicine, 4th Medical Discipline, “Iuliu Hațieganu” University of Medicine and Pharmacy, 400015 Cluj-Napoca, Romania; borbei_rahela@yahoo.ro (R.B.); saracut_ioana_raluca@elearn.umfcluj.ro (I.D.); ceterasligia@yahoo.com (L.-M.C.); mihai.clim07@gmail.com (M.C.); teodora.alexescu@umfcluj.ro (T.-G.A.); vasile.milaciu@umfcluj.ro (M.-V.M.); albmirela@yahoo.ro (M.-G.P.); andreea.ceza.irimie@elearn.umfcluj.ro (C.-A.G.); angelacozma@yahoo.com (A.C.); hilda.orasan@umfcluj.ro (O.-H.O.); 2 Department of Gastroenterology, Regional Institute of Gastroenterology and Hepatology “Prof. Dr. Octavian Fodor”, 400394 Cluj-Napoca, Romania; 3 Department of Medical Biochemistry, Faculty of Medicine, “Iuliu Hațieganu” University of Medicine and Pharmacy, 400349 Cluj-Napoca, Romania; lprocopciuc@umfcluj.ro

**Keywords:** metabolic syndrome, pancreatic cancer, insulin resistance, adipokines, oxidative stress, gut microbiome, early detection, biomarkers

## Abstract

Metabolic syndrome (MetS) is a composite classification defined by heterogeneous combinations of cardiometabolic abnormalities. Several components have been associated with pancreatic cancer (PC), but studies often conflate long-term metabolic exposure, tumor-related prediagnostic metabolic change, and prognosis after diagnosis. This review critically evaluates these three settings. This narrative review searched PubMed, Scopus, and Web of Science through April 2026, prioritizing meta-analyses, prospective cohorts, Mendelian randomization studies, clinical studies, preclinical mechanistic evidence, and guidelines. Evidence was interpreted with attention to reverse causation, residual confounding, outcome definitions, and validation. Meta-analyses report a 25–34% higher PC risk among individuals with MetS, with a stepwise gradient across the number of metabolic components; hyperglycemia showed the strongest association among individual components (RR 1.55, 95% CI 1.42–1.70). However, MetS represents multiple versions of exposure, and the epidemiological and clinical evidence remains predominantly observational. Proposed mechanisms involving insulin signaling, adipokines, oxidative stress, epigenetic regulation, and the microbiome are largely preclinical and are not pancreas-specific. New-onset diabetes, rising HbA1c, and involuntary weight loss may mark occult pancreatic ductal adenocarcinoma, but available risk-enrichment models and biomarkers are insufficiently validated for routine screening. After diagnosis, metabolic status and body-composition measures have been associated with survival and perioperative outcomes, although confounding and cancer-related cachexia complicate interpretation. MetS is consistently associated with PC risk but is neither a single causal exposure nor an established target for PC prevention. MetS alone does not justify pancreatic imaging or biomarker testing. Lifestyle and pharmacologic treatment should follow established cardiometabolic indications; their effects on PC incidence remain unproven. Priorities include component-specific causal studies and prospective validation of risk-enrichment strategies.

## 1. Introduction

Pancreatic ductal adenocarcinoma (PDAC) accounts for approximately 85% of pancreatic malignancies and remains one of the most lethal solid tumors because of its late clinical presentation, limited surgical eligibility, and resistance to systemic therapy [1]. Non-ductal pancreatic malignancies—including pancreatic neuroendocrine neoplasms and acinar cell carcinoma—differ from PDAC in their biology, prognosis, and risk-factor profiles [1,2]. In the United States, pancreatic cancer (PC) has been projected to become the second leading cause of cancer-related mortality by 2030 [3].

The global prevalence of metabolic syndrome (MetS) has more than doubled during the past two decades, with an estimated 1.54 billion adults affected in 2023 [4]. Its relationship with obesity-related cancers has consequently received increasing attention [5]. MetS is commonly operationalized as the presence of at least three of five abnormalities: central adiposity, hyperglycemia, elevated blood pressure, hypertriglyceridemia, and reduced high-density lipoprotein cholesterol [6,7]. However, it is a classification rather than a single biological exposure. Two individuals may meet the diagnostic criteria through largely non-overlapping combinations of abnormalities, with potentially different underlying mechanisms and management requirements. Consequently, an association between MetS and cancer cannot automatically be interpreted as the effect of a single modifiable entity.

Several components of MetS, particularly hyperglycemia and central adiposity, have been associated with PC. Meta-analyses have also reported higher PC risk among individuals classified as having MetS [8,9], while population-based data suggest that metabolic status is associated with risk across body-weight categories [10]. Interpretation remains difficult because most evidence is observational and because the temporal relationship is bidirectional: long-standing metabolic abnormalities may precede and potentially contribute to carcinogenesis, whereas occult PDAC can induce new-onset diabetes, worsening glycemic control, and weight loss months or years before clinical diagnosis [11,12].

The broad topic of “metabolic syndrome and pancreatic cancer” therefore encompasses three temporally and causally distinct questions: whether long-standing metabolic dysfunction contributes to the development of PDAC; whether metabolic changes preceding diagnosis are consequences or markers of occult disease; and whether metabolic status and body composition are associated with outcomes after diagnosis. These questions require different study designs, differ in their vulnerability to reverse causation and residual confounding, and would have different clinical implications. Failure to distinguish these settings can make observational associations appear more clinically actionable than the evidence permits, particularly with respect to pancreatic cancer screening and pharmacologic prevention.

Previous reviews and consensus papers have generally examined obesity, diabetes, insulin resistance, or individual metabolic pathways [13,14], or have summarized epidemiological associations without consistently separating these temporal settings. The present review differs in two respects. First, it evaluates etiologic, prediagnostic, and prognostic evidence separately, explicitly distinguishing association from causality and risk prediction from early detection. Second, it considers conventional metabolic factors alongside emerging risk-enrichment models, biomarkers, and multi-omics signatures, not to recommend their current clinical use but to identify the validation requirements that would need to be met before implementation.

Biological mechanisms are considered first as a potential shared substrate for these three settings, while acknowledging that most mechanistic evidence is preclinical and not specific to the pancreas. The aim is to provide a critical narrative synthesis of the available evidence rather than to propose MetS-based pancreatic cancer screening or treatment strategies.

## 2. Materials and Methods

This article was designed as a critical narrative review and was not intended to provide a systematic or exhaustive synthesis of all available evidence. No review protocol was registered. PubMed/MEDLINE, Scopus, and Web of Science were searched from database inception through April 2026. Searches were iterative and combined terms related to metabolic syndrome and its components (“metabolic syndrome,” “insulin resistance,” “obesity,” “central adiposity,” “hyperglycemia,” “diabetes,” “dyslipidemia,” and “hypertension”) with terms related to pancreatic malignancy (“pancreatic cancer” and “pancreatic ductal adenocarcinoma”) and the principal domains of the review (“carcinogenesis,” “risk,” “prognosis,” “survival,” “body composition,” “new-onset diabetes,” “early detection,” “biomarkers,” and “multi-omics”). Search terms and combinations were adapted to the syntax of each database and refined as additional concepts and relevant studies were identified. Reference lists of included publications were also screened manually.

Literature searching and study selection were performed by two authors (R.B. and V.D.B.), with disagreements resolved by discussion, and an exact record count and a PRISMA flow diagram were not produced. This approach reflects the narrative design of the review and represents an important limitation regarding reproducibility and completeness.

Study selection was guided by relevance to the three settings defined: long-standing metabolic exposure and PC risk, prediagnostic metabolic change as a potential marker of occult disease, and metabolic status or body composition as prognostic factors after diagnosis. For epidemiological associations, priority was given to systematic reviews, meta-analyses, large prospective cohorts, and Mendelian randomization studies. Studies of early detection were prioritized when they included longitudinal prediagnostic measurements, external validation, or clinically relevant performance measures. Prognostic evidence was preferentially drawn from multivariable-adjusted clinical cohorts, while randomized trials and clinical guidelines were prioritized when evaluating lifestyle or pharmacologic interventions. Preclinical studies were included when they provided mechanistic information not available from human studies. Case reports, non-peer-reviewed conference abstracts, and non-English-language publications were excluded.

No new quantitative synthesis was performed, and effect estimates were reported as presented in the cited publications. No formal risk-of-bias or evidence-certainty instrument was applied. Evidence was interpreted according to study design, adjustment for confounding, vulnerability to reverse causation, temporal relationship to PC diagnosis, and degree of external or prospective validation. Consequently, the summarized evidence categories are descriptive rather than formal GRADE certainty ratings. These methodological features should be considered when interpreting the conclusions of this narrative review.

### Terminology

The abbreviation PDAC is used when ductal histology was confirmed or when non-ductal pancreatic malignancies were explicitly excluded. PC is used when studies reported pancreatic cancer without histological confirmation, particularly in registry- or administrative-data analyses. Estimates described as PC should therefore not be assumed to be histologically specific to PDAC. 

In this review, risk prediction refers to estimation of the probability of a future PC diagnosis, whereas early detection refers to attempts to identify an occult malignancy that is already present. Screening denotes systematic testing of asymptomatic individuals, while surveillance is reserved for structured programs involving people with established hereditary or familial risk. Clinical vigilance refers to heightened attention to atypical findings—such as new-onset diabetes accompanied by involuntary weight loss—during routine clinical care and does not imply a validated surveillance program.

## 3. Biological Pathways Linking MetS Components to Pancreatic Carcinogenesis

**Figure 1:**
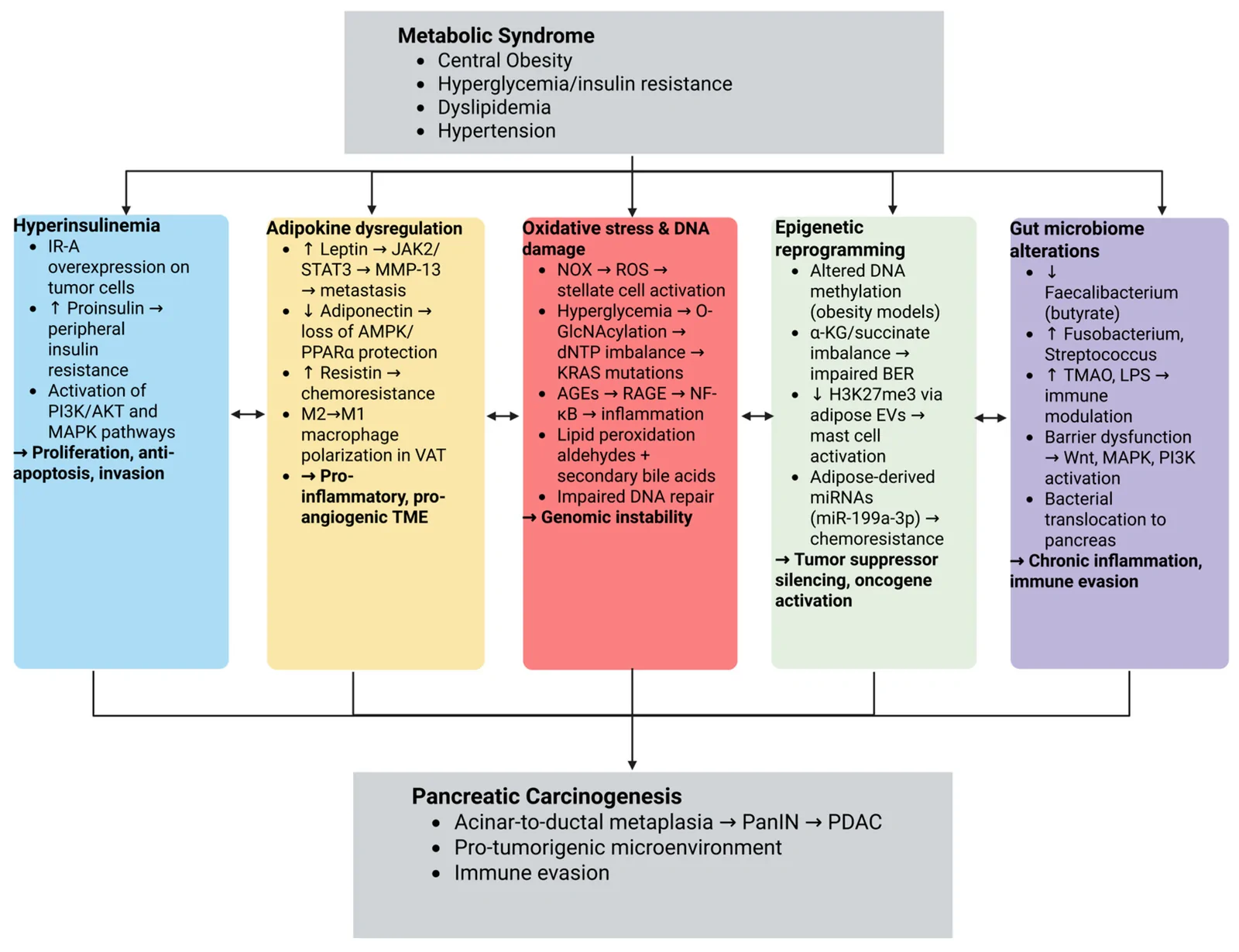
Proposed biological pathways through which individual MetS components may contribute to pancreatic carcinogenesis. Hyperinsulinemia promotes cell-proliferation, anti-apoptosis mechanisms and invasion. Adipokine dysregulation has a proinflammatory and pro-angiogenic effect within the tumor microenvironment. Oxidative stress and DNA damage lead to genomic instability. Epigenic reprogramming leads to tumor suppressor silencing and oncogene activation. Gut microbiome alteration are responsible for chronic inflammation and immune evasion. Most supporting evidence is preclinical and does not establish causality in humans. Arrows indicate the presumed direction of the causal relationship. Abbreviations: IR-A, insulin receptor isoform A; PI3K, phosphatidylinositol 3-kinase; AKT, protein kinase B; MAPK, mitogen-activated protein kinase; JAK2, janus kinase 2; STAT3, signal transducer and activator of transcription 3; MMP-13, matrix metalloproteinase-13; AMPK, AMP-activated protein kinase; PPARα, peroxisome proliferator-activated receptor alpha; VAT, visceral adipose tissue; TME, tumor microenvironment; NOX, NADPH oxidase; ROS, reactive oxygen species; O-GlcNAcylation, O-linked β-N-acetylglucosamine modification; dNTP, deoxyribonucleotide triphosphates; KRAS, Kirsten rat sarcoma viral oncogene homolog; AGEs, advanced glycation end products; RAGE, receptor for advanced glycation end products; NF-κB, Nuclear Factor Kappa B; BER, base excision repair; H3K27me3, trimethylation of histone H3 lysine 27; EVs, extracellular vesicles; miRNA, microRNA; LPS, lipopolysaccharide; TMAO, trimethylamine N-oxide; PanIN, pancreatic intraepithelial neoplasia; PDAC, pancreatic ductal adenocarcinoma.

Several interrelated biological pathways have been proposed to explain how metabolic abnormalities commonly grouped within MetS might influence pancreatic carcinogenesis: chronic hyperinsulinemia, adipokine dysregulation, oxidative stress and genomic instability, epigenetic reprogramming, and gut microbiome alterations. These processes may operate concurrently and contribute to biological conditions that favor acinar-to-ductal metaplasia, PanIN formation, or tumor progression (Figure 1). Most supporting evidence, however, derives from in vitro studies, animal models, or mechanistic observations across several obesity-related malignancies, and is therefore reviewed below as a source of biological plausibility rather than of causal inference.

### 3.1. Insulin Resistance and Hyperinsulinemia

Insulin resistance is frequently accompanied by compensatory hyperinsulinemia, which increases the exposure of peripheral and pancreatic tissues to insulin. In addition to its metabolic effects, insulin can activate mitogenic signaling through the insulin receptor (IR). Of the two principal IR isoforms, IR-A has comparatively greater mitogenic activity, whereas IR-B more prominently mediates metabolic responses; a relative predominance of IR-A has been described in several cancers [15]. This observation is not specific to PDAC. More pancreas-specific evidence comes from a Kras-mutant mouse model of diet-induced hyperinsulinemia, in which deletion of insulin receptors from pancreatic acinar cells attenuated digestive-enzyme synthesis, local inflammation, and PanIN formation [16]. Although mechanistically informative, this evidence remains preclinical.

Signaling through the insulin and IGF-1 receptors engages the PI3K/AKT and MAPK/ERK networks, which regulate metabolism, proliferation, survival, and apoptosis [17,18,19,20,21]. These networks are frequently activated in PDAC, including downstream of oncogenic KRAS, which is mutated in more than 90% of tumors [17,18,22]. Increased AKT activity attributable to hyperphosphorylation has been reported in approximately 60% of PDAC samples [17]. The PI3K/AKT network has also been implicated in MetS-related metabolic abnormalities [23], whereas JNK, ERK, and p38 MAPKs contribute to inflammation-associated insulin resistance across adipose tissue, pancreatic β-cells, the liver, and skeletal muscle [24]. This overlap provides a potential point of convergence between metabolic and tumor-intrinsic signaling. However, because these pathways are widely used across normal tissues and multiple malignancies, their activation is not specific to a MetS–PDAC sequence.

Experimental studies have examined different stages of pancreatic neoplasia. MAPK signaling was required for acinar-cell dedifferentiation and PanIN development in Kras-driven mouse models [25]. In models of established pancreatic cancer, physiological insulin concentrations increased glucose utilization and subsequently stimulated tumor-cell proliferation through MAPK and PI3K signaling [26]. Separately, high-glucose conditions enhanced the invasive capacity of pancreatic cancer cells through the SOD2/H_2_O_2_/ERK/p38 axis in cultured cells and in a diabetic mouse xenograft model [27].

Directionality is further complicated by evidence that PDAC can itself promote peripheral insulin resistance and worsening glycemic control through tumor-derived mediators [28]. Hyperinsulinemia observed near diagnosis may therefore represent long-standing metabolic dysfunction, a consequence of occult disease, or both. The most direct pancreas-specific evidence linking hyperinsulinemia to tumor initiation comes from animal models, and these data do not establish that lowering insulin levels or correcting insulin resistance would prevent PDAC in humans.

### 3.2. Adipokine Dysregulation

Obesity alters both the endocrine and immune functions of adipose tissue. Circulating leptin concentrations generally increase with adiposity, whereas adiponectin concentrations tend to decrease, and expansion of visceral adipose tissue is accompanied by chronic low-grade inflammation [29,30,31]. Adiponectin enhances insulin sensitivity through pathways involving AMPK and PPARα and has anti-inflammatory properties [29,32]. Its relationship with pancreatic cancer appears complex: observational studies have reported inverse, positive, or null associations between circulating adiponectin and PC risk, with differences in study populations, adiposity, and diabetes potentially contributing to this heterogeneity [30,32]. Timing of blood collection is a distinct concern because cancer-associated weight loss and cachexia can alter circulating adipokine concentrations. Samples obtained at or near diagnosis may therefore reflect occult disease as well as the antecedent metabolic state. Experimental findings are similarly context-dependent: adiponectin signaling reduced proliferation through attenuation of GSK-3β/β-catenin signaling in some human pancreatic cancer cell and xenograft models, whereas other murine models suggested pro-survival effects [32].

Leptin can activate PI3K/AKT, JAK2/STAT3, and other signaling pathways involved in proliferation, metabolism, and cell migration. In pancreatic cancer models, leptin promoted tumor-cell proliferation and invasive behavior, whereas adiponectin and the adiponectin-receptor agonist AdipoRon inhibited leptin-induced STAT3 signaling and reduced tumor growth [33]. In one study, leptin increased MMP-13 expression through JAK2/STAT3 signaling and enhanced invasion and metastasis in experimental models; in human pancreatic tumor specimens, expression of the leptin receptor and MMP-13 was associated with lymph-node metastasis, providing a clinical correlate for the experimental findings [34].

Expansion of visceral adipose tissue is accompanied by macrophage recruitment and remodeling toward more pro-inflammatory states, with increased production of mediators including IL-6, IL-1β, TNF-α, and MCP-1 [31]. These mediators can influence transformed pancreatic epithelial cells and other components of the tumor microenvironment. Adipocyte-derived factors and lipids may also support tumor-cell metabolism, whereas established tumors can promote adipocyte lipolysis, dedifferentiation, and systemic changes in body composition [31,35]. This bidirectional communication appears more relevant to tumor progression and cancer-associated wasting than to tumor initiation and connects mechanistically with the body-composition changes.

Angiogenic signaling represents another potential point of convergence. VEGF expression has been investigated in relation to PDAC progression, treatment resistance, and prognosis [36,37]. Separately, circulating VEGF concentrations were correlated with visceral fat area in a small study of overweight and obese individuals without cancer [38], while leptin has demonstrated proangiogenic effects in experimental systems through stimulation of VEGF-related signaling [30]. Together, these observations suggest that visceral adiposity and adipokine signaling may contribute to an angiogenic environment, although the connection has not been demonstrated as a continuous causal pathway in PDAC.

Taken together, adipokine signaling, adipose-tissue inflammation, and metabolic exchange between adipocytes and tumor cells provide biologically plausible links between obesity-related metabolic dysfunction and pancreatic neoplasia. Pancreas-specific evidence remains predominantly experimental, while human studies of circulating adipokines are heterogeneous; consequently, neither adiponectin nor leptin currently represents a validated biomarker or a clinically established target for PDAC prevention.

### 3.3. Oxidative Stress and Genomic Instability

Oxidative stress arises when the production of reactive oxygen species (ROS) exceeds cellular antioxidant capacity. Excess ROS can modify DNA bases, induce single- and double-strand breaks, and contribute to chromosomal instability. Hyperglycemia, dyslipidemia, and hypertension have each been associated with altered redox homeostasis in MetS, although the predominant sources and downstream effects vary among tissues [39,40]. In pancreatic disease models, nicotinamide adenine dinucleotide phosphate oxidase (NOX) enzymes represent one source of ROS and have been implicated in acinar-cell injury, pancreatic stellate-cell activation, macrophage polarization, inflammation, and fibrosis [41]. These are generic responses to pancreatic injury rather than a tumor-specific pathway.

Experimental pancreatic-cell systems provide a more specific potential connection between hyperglycemia and genome instability. In non-tumorigenic pancreatic cells, sustained high-glucose exposure increased O-GlcNAcylation of phosphofructokinase-1 and the RRM1 subunit of ribonucleotide reductase, disrupted nucleotide balance, increased DNA damage, and favored the acquisition of KRAS mutations [42]. Whether the same sequence occurs during human PDAC initiation remains unknown. Chronic hyperglycemia also promotes the formation of advanced glycation end products (AGEs), while signaling through the receptor for AGEs (RAGE) can sustain inflammatory and KRAS-dependent pathways in experimental pancreatic cancer models [13]. In this context, RAGE is more appropriately considered a modulator of oncogenic signaling than evidence that AGE accumulation generates KRAS mutations.

Obesity-associated oxidative and inflammatory stress may also generate reactive lipid-peroxidation products and alter exposure to bile-acid metabolites, both of which act as mutagens in experimental settings [43], and has separately been associated with impaired DNA-damage responses and repair capacity [44]. Most of this evidence derives from non-pancreatic tissues and experimental models and therefore supports a general model of obesity-related genomic vulnerability rather than direct demonstration of obesity-induced mutagenesis in the human pancreas.

Mitochondrial dysfunction provides an additional connection between metabolic stress and redox signaling. In Kras-mutant acinar-cell models, oncogenic KRAS altered mitochondrial metabolism and increased ROS production, activating EGFR-dependent signaling involved in acinar-cell dedifferentiation and the formation of pancreatic precursor lesions [45]. In this experimental setting, mitochondrial ROS were downstream of oncogenic KRAS rather than evidence that metabolic dysfunction had initiated the mutation. At the systemic level, obesity and MetS have been associated with excess substrate availability, impaired mitochondrial oxidative metabolism, and increased ROS production, while mitochondrial and endoplasmic-reticulum stress in hypertrophic adipocytes can reinforce inflammatory signaling and altered adipokine secretion [46,47,48]. Mitochondrial alterations may also be relevant after malignant transformation: somatic mitochondrial DNA variants accumulate during PDAC progression, and variants affecting the ND4 and ND6 genes were associated with shorter overall survival [49], an observation concerning tumor evolution and prognosis rather than metabolic initiation.

Taken together, oxidative stress, altered nucleotide metabolism, and mitochondrial dysfunction provide plausible points of interaction between metabolic abnormalities and pancreatic neoplasia. The strongest pancreas-specific evidence is experimental and supports several possible directions of effect, including metabolic stress preceding neoplasia, oncogenic KRAS generating oxidative stress, and mitochondrial alterations accompanying tumor progression—none of which establishes oxidative stress as a validated target for PDAC prevention.

### 3.4. Emerging Molecular Links

#### 3.4.1. Non-Coding RNAs, Epigenetic Regulation, and Extracellular Vesicles

Adipose tissue-derived microRNAs (miRNAs) participate in adipocyte differentiation, insulin sensitivity, and inflammatory signaling, while obesity can alter circulating and extracellular-vesicle miRNA profiles [50,51]. These general observations have led to the hypothesis that adipose-derived miRNAs may convey signals between metabolic tissues and tumors. In a PDAC-specific experimental study, cancer-associated adipocytes transferred exosomal miR-199a-3p to pancreatic cancer cells, suppressing SOCS7 and activating the STAT3/SAA1 network, with consequent increases in proliferation, motility, and chemoresistance [52]. Because the adipocytes in this model were already conditioned by the tumor, the findings are more directly relevant to progression of established pancreatic cancer than to metabolically driven tumor initiation.

Metabolic state can influence epigenetic regulation because metabolites serve as substrates or cofactors for chromatin-modifying enzymes, while epigenetic changes can reciprocally alter metabolic gene expression. A recent synthesis examined this relationship in colorectal cancer [53], whereas pancreatic premalignancy has also been associated with changes in DNA methylation, histone regulation, chromatin organization, and non-coding RNA expression [54]. In mice fed a high-fat diet, pancreatic exocrine cells displayed altered DNA-methylation patterns involving genes related to cell growth and DNA repair [55]. More recently, experiments combining high-fat-diet-fed Kras-mutant mice with KRAS-mutated human pancreatic ductal epithelial cells identified a reduction in the α-ketoglutarate/succinate ratio, impaired thymine DNA glycosylase activity and base-excision repair, and increased susceptibility to pancreatic tumorigenesis [56]. These studies provide a pancreas-specific experimental connection between dysmetabolism and epigenetic regulation, although equivalent longitudinal evidence in humans is not yet available.

Established PDACs also display promoter methylation and histone modifications affecting tumor-suppressive and oncogenic programs [57]. A separate experimental study showed that extracellular vesicles from obese visceral adipose tissue delivered cathepsin A to PDAC cells, resulting in pseudouridine production, mast-cell activation, suppression of CD8+ T-cell activity, and resistance to immune checkpoint blockade [58]. This adipose tissue–tumor communication pathway is primarily relevant to tumor progression and treatment response.

#### 3.4.2. Microbiome-Related Hypotheses

Obesity, diet, and metabolic dysfunction can alter gut microbial composition and intestinal permeability, thereby changing exposure to microbial products capable of influencing systemic inflammation and tumor-associated signaling [59]. However, evidence connecting these changes specifically to pancreatic carcinogenesis remains heterogeneous. A meta-analysis of 10 case–control studies found no significant difference in overall microbial richness or α-diversity between patients with PC and controls, although differences were reported in the relative abundance of several bacterial genera, including lower abundance of *Bacteroides*, *Prevotella*, and *Actinomyces* and higher abundance of *Fusobacterium*, *Streptococcus*, and *Escherichia–Shigella* [60]. Because samples were generally obtained at or after diagnosis, these patterns may reflect the cancer itself, biliary or pancreatic dysfunction, dietary changes, medication exposure, or treatment rather than an antecedent cause.

A subsequent analysis combining cross-sectional sequencing, Mendelian randomization, and metabolite mediation identified several microbial taxa and circulating metabolites associated with PC risk, but some genetic associations were discordant with the microbial patterns observed in patients with established disease [61]. Short-chain fatty acid-producing organisms and their metabolites have been proposed to support epithelial integrity and antitumor immune function, but much of this evidence derives from experimental systems or malignancies other than PDAC [62]. Other microbial products may have context-dependent effects: trimethylamine N-oxide has enhanced antitumor immunity in preclinical PDAC models, whereas lipopolysaccharide-related signaling can promote inflammation and immune remodeling [63]. These findings do not support classifying microbial metabolites collectively as either tumor-promoting or tumor-suppressive.

Microbial material has been detected in some pancreatic tumors, and anatomical or hematogenous translocation from the oral cavity or intestine has been proposed as a potential origin of the intratumoral microbiome [64]. Tumor-resident microorganisms may influence drug metabolism, inflammatory signaling, and antitumor immunity, but the routes of colonization and their temporal relationship to tumor development remain incompletely established.

Taken together, non-coding RNAs, epigenetic regulation, extracellular vesicles, and microbial factors represent candidate mechanisms rather than a unified molecular pathway connecting MetS to PDAC. Their temporal significance also differs: high-fat-diet and Kras-mutant models primarily inform hypotheses concerning tumor initiation, whereas cancer-associated adipocytes, extracellular vesicles from obese visceral adipose tissue, and the intratumoral microbiome appear more relevant to progression or treatment response after malignancy has developed. These distinctions are essential when interpreting the corresponding etiologic, prediagnostic, and prognostic evidence.

## 4. Epidemiological Evidence Linking MetS to PC

### 4.1. Metabolic Syndrome as a Composite Risk Factor

Metabolic syndrome is a classification rather than a single exposure. It is defined by different combinations of hyperglycemia, central adiposity, hypertension, hypertriglyceridemia, and reduced HDL cholesterol, so that two individuals may meet the same criteria through largely non-overlapping abnormalities and would be managed differently. Composite estimates therefore correspond to multiple versions of the exposure, and any intervention implied by them is correspondingly ill-defined: “treating” or “resolving” metabolic syndrome is not a single procedure. The pooled estimates below are reported because they describe how risk varies with accumulated metabolic burden, not because they identify a modifiable entity. The causal question of interest is whether intervening on a specified component—glycemia, visceral adiposity, blood pressure, lipid metabolism, or insulin resistance—alters pancreatic cancer incidence, and the studies reviewed here were not designed to answer it.

MetS is consistently associated with an increased risk of PC, with pooled estimates ranging from RR 1.25 (95% CI 1.20–1.30) [9] to RR 1.34 (95% CI 1.23–1.46, *p* < 0.0001, I^2^ = 38.8%) [8]. Risk increases progressively with the number of components present, from HR 1.11 (95% CI 1.09–1.12) for one to HR 2.03 (95% CI 1.90–2.17) for five [65]. Metabolic status is associated with risk across body-weight categories: risk was elevated in metabolically unhealthy normal-weight individuals (HR 1.52) and in metabolically unhealthy individuals with obesity (HR 1.34) relative to a metabolically healthy normal-weight reference, with no statistically significant association observed among metabolically healthy individuals with obesity [10]. Individuals whose MetS resolved during follow-up had lower subsequent risk than those with persistent MetS (HR 1.12, 95% CI 1.04–1.21 vs. HR 1.30, 95% CI 1.23–1.37, both against the same reference group) [66]. The association is consistently stronger in women (RR 1.64, 95% CI 1.41–1.90) than in men (RR 1.26, 95% CI 1.03–1.54) [8], and the Me-Can Project, analyzing approximately 580,000 participants, found the composite MetS z-score associated with PC risk in women but not in men [67].

These estimates share a set of limitations that constrain their interpretation. Exposure and outcome were defined by administrative codes without histological confirmation, so the narrow confidence intervals reflect cohort size rather than precision of case ascertainment. Whether the gradient across metabolic phenotypes reflects a genuine difference in etiology or differential detection and classification cannot be determined from these data. Recovery from MetS was a post-baseline, time-varying exposure and is therefore susceptible to healthy-user effects and residual confounding; it should not be interpreted as the effect of deliberately treating MetS. The sex difference is reproduced across independent datasets but its basis is unresolved, and it has not been examined in relation to differences in fat distribution, hormonal exposure, or diagnostic pathways.

### 4.2. Individual Components of MetS

The individual components of MetS do not show equivalent associations with PC risk. Although exposure definitions and adjustment strategies varied among the contributing studies, hyperglycemia showed the largest pooled association (RR 1.55, 95% CI 1.42–1.70), followed by reduced high-density lipoprotein cholesterol (HDL-C; RR 1.24, 95% CI 1.11–1.38) and hypertension (RR 1.10, 95% CI 1.01–1.19). By contrast, the pooled estimates for obesity (RR 1.13, 95% CI 0.96–1.32) and hypertriglyceridemia (RR 0.96, 95% CI 0.87–1.07) were not statistically significant, although the confidence interval for obesity remained compatible with a modest association [8] (Table 1). These findings indicate heterogeneity among the components rather than a uniform effect of the MetS classification.

The temporal relationship is particularly important for diabetes. In a meta-analysis of 44 studies, diabetes of at least 2 years’ duration was associated with PC risk (RR 1.64, 95% CI 1.52–1.78); the pooled estimates were RR 1.58 (95% CI 1.42–1.75) for diabetes of at least 5 years’ duration and RR 1.50 (95% CI 1.28–1.75) for diabetes persisting for at least 10 years [71]. The persistence of an association after a long duration is compatible with an etiologic contribution, whereas its attenuation with increasing duration also illustrates the influence of cases occurring closer to diabetes onset, some of which may reflect occult pancreatic malignancy. Diabetes developing shortly before PC diagnosis should therefore be interpreted separately as a potential prediagnostic manifestation of disease.

Prediagnostic biomarkers provide additional evidence concerning insulin-related pathways. In a nested case–control analysis across five prospective cohorts, participants in the highest versus lowest quintiles of HbA1c, insulin, and proinsulin had ORs of 1.79 (95% CI 1.17–2.72), 1.57 (95% CI 1.08–2.30), and 2.22 (95% CI 1.50–3.29), respectively [72]. Associations with insulin and proinsulin were stronger for cancers diagnosed at least 10 years after blood collection. When HbA1c, insulin, and proinsulin were included simultaneously, only proinsulin remained statistically significant (OR 2.55, 95% CI 1.54–4.21), a pattern more consistent with insulin resistance and compensatory hyperinsulinemia than with hyperglycemia alone [72].

The association with HDL-C is less robust. In addition to the pooled estimate reported above, a population-based longitudinal cohort found that a change from normal to low HDL-C was associated with PC incidence compared with persistently normal HDL-C (adjusted HR 1.11, 95% CI 1.05–1.17), with the association observed predominantly among participants younger than 60 years [73]. However, the association was no longer statistically significant in the more stringent sensitivity analysis that excluded cancers diagnosed before the second HDL-C assessment. Reduced HDL-C may therefore represent a metabolic correlate of risk or evolving disease rather than an established etiologic factor.

Evidence concerning adiposity differs according to study design. A two-sample Mendelian randomization analysis found that a genetically predicted one-standard-deviation increase in BMI was associated with PDAC risk (OR 1.34, 95% CI 1.09–1.65), as was genetically predicted fasting insulin (OR 1.66, 95% CI 1.05–2.63), whereas genetic liability to type 2 diabetes and dyslipidemia was not associated with risk [68]. A subsequent analysis reported a nominally significant association for genetically predicted BMI-adjusted waist-to-hip ratio, but not BMI; the waist-to-hip ratio result was not reproduced by the other MR sensitivity methods and was attenuated after accounting for type 2 diabetes [69]. Observational estimates may also be biased downward by cancer-related weight loss before diagnosis. An umbrella review found that only 5 of 13 previous evidence syntheses had explicitly addressed this problem and that effect estimates generally increased when longer periods of early follow-up were excluded [74].

More recent MR evidence from a Japanese population found that genetic liability to type 2 diabetes was associated with pancreatic cancer risk (OR 1.16, 95% CI 1.08–1.25 per one-unit increase in the log odds of diabetes), including after adjustment for genetically predicted BMI (OR 1.15, 95% CI 1.07–1.24), whereas genetically predicted BMI was not associated with risk (OR 1.00, 95% CI 0.95–1.04 per 1 kg/m^2^) [70]. Differences between these genetic analyses may reflect population ancestry, phenotype definitions, instrument selection, or statistical power. Overall, the component-specific evidence is most consistent with a role for long-standing insulin-related dysfunction and adiposity, while associations with HDL-C and hypertension are smaller and hypertriglyceridemia has not shown a pooled association. None of these findings establishes that modifying a particular component will reduce PC incidence.

### 4.3. Methodological Challenges

Interpretation of the epidemiological association between MetS and PC is constrained by several methodological issues, most of which apply to the estimates presented above.

Reverse causation is the central concern. Occult pancreatic tumors can disrupt glucose homeostasis and induce catabolic weight loss months or years before clinical diagnosis, thereby biasing metabolic exposures in different directions: prediagnostic hyperglycemia and recently diagnosed diabetes may produce stronger positive associations, whereas tumor-related weight loss may attenuate associations with BMI or obesity [11,12]. Excluding cancers diagnosed during early follow-up or stratifying analyses according to the interval between metabolic measurement and diagnosis can reduce this bias but cannot eliminate it. Where such approaches have been applied, associations with insulin and proinsulin persisted or became stronger after a latency of at least 10 years [72], while estimates for adiposity generally increased when longer periods of early follow-up were excluded [74].

Ascertainment and outcome classification introduce separate uncertainties. Individuals with diabetes or other metabolic abnormalities may have more frequent contact with healthcare services and may consequently undergo more diagnostic investigations, potentially increasing the likelihood that pancreatic malignancy is detected. The magnitude of this surveillance effect has not been quantified in the available studies. In administrative-database analyses, PC was identified using diagnostic or reimbursement codes without consistent histological confirmation [65,66]. Such studies cannot fully distinguish differential cancer incidence from differential detection or coding, and their outcomes should not automatically be interpreted as histologically confirmed PDAC.

Residual confounding also remains difficult to exclude. Smoking and heavy alcohol consumption are associated with PC risk and may cluster with metabolic abnormalities, while diet, physical activity, socioeconomic circumstances, and healthcare use are measured and adjusted for inconsistently. Associations with obesity, central adiposity, and diabetes have also been reported among postmenopausal women who had never smoked and did not consume alcohol, suggesting that the metabolic signal is not explained entirely by these two exposures; however, restriction to this selected population does not eliminate other sources of confounding and limits generalizability [75]. Chronic pancreatitis adds further complexity because it is a strong risk factor for pancreatic malignancy and may share determinants with metabolic dysfunction. Depending on the temporal and causal structure, it may act as a confounder, an intermediate factor, or part of an evolving pancreatic disease process; inconsistent adjustment can therefore result in either residual confounding or overadjustment [12].

Exposure heterogeneity is another important limitation. No single MetS definition has been adopted universally, and the contributing studies applied NCEP/ATP III, Japanese, Korean, and other modified criteria to populations with different distributions of the underlying abnormalities. Moreover, MetS status and its individual components were often assessed only once, despite their potential to change during follow-up. Pooled estimates therefore combine studies in which the same diagnostic label may represent different combinations, severities, and durations of metabolic abnormalities.

Mendelian randomization reduces some forms of conventional confounding and reverse causation but introduces different assumptions concerning instrument validity, horizontal pleiotropy, ancestry, and the interpretation of lifelong genetic liability. The available analyses have not converged on a single dominant metabolic exposure: associations have differed for BMI, central adiposity, fasting insulin, and genetic liability to type 2 diabetes across populations and analytical methods [68,69,70]. These estimates cannot be interpreted as the expected effect of modifying the corresponding factor later in life. No interventional study has established that correction of MetS or any individual metabolic abnormality reduces PC incidence.

## 5. Prediagnostic Metabolic Changes as Markers of Occult PDAC

### 5.1. New-Onset Diabetes as a Manifestation of Occult Disease

Long-standing diabetes is associated with a modest increase in PC risk, whereas diabetes of recent onset shows a substantially stronger short-term association. In a population-based cohort, compared with individuals without diabetes, the HR for PC was 3.81 (95% CI 2.97–4.88) among those with new-onset diabetes and 1.53 (95% CI 1.11–2.11) among those with long-standing diabetes [11,76]. The concentration of risk within the first three years after diabetes diagnosis is consistent with a bidirectional relationship: long-standing diabetes may represent an antecedent metabolic exposure, whereas diabetes developing close to cancer diagnosis may, in some patients, be caused or accelerated by an occult pancreatic malignancy.

Accordingly, in the prediagnostic setting, new-onset diabetes is more appropriately evaluated as a potential marker of already-present disease than as evidence of a carcinogenic effect operating over a short interval. However, because only a small proportion of individuals with new-onset diabetes are diagnosed with PC, diabetes onset alone is insufficient for early detection; its clinical value depends on further enrichment using additional demographic, metabolic, or clinical features.

### 5.2. Prediagnostic Trajectories of HbA1c and Body Weight

Metabolic changes may become detectable several years before PC diagnosis. In a matched case–control analysis of UK primary-care records that included 8777 patients with PC and 34,979 matched controls, the association with increasing HbA1c remained statistically significant up to 3 years before diagnosis, whereas the association with declining BMI remained significant up to 2 years before diagnosis. During the year immediately preceding diagnosis, each 1 mmol/mol increase in HbA1c was associated with an adjusted OR of 1.06 (95% CI 1.06–1.07), and each 1 kg/m^2^ decrease in BMI was associated with an adjusted OR of 1.05 (95% CI 1.05–1.06) [77]. The pattern differed according to pre-existing diabetes status: the association with increasing HbA1c was stronger among individuals without diabetes, whereas the association with declining BMI was stronger among those with established diabetes [77]. These group-level associations were derived from opportunistically recorded primary-care measurements and do not constitute validated individual diagnostic thresholds.

The 2025 ADA Standards of Care note that atypical new-onset diabetes—particularly in a middle-aged or older person with a lean body habitus and no family history of diabetes—may precede the diagnosis of pancreatic adenocarcinoma. However, routine PC screening is not recommended in the absence of additional features such as involuntary weight loss or abdominal pain [78]. This guidance supports clinical vigilance when metabolic trajectories and other warning features occur together, but not systematic imaging based on hyperglycemia alone.

### 5.3. Risk-Enrichment Tools in New-Onset Diabetes

The development of diabetes after the age of 50, especially when accompanied by involuntary weight loss, a lean phenotype, or absence of a family history of type 2 diabetes, may raise suspicion for an underlying pancreatic malignancy [11,78]. Population-based cohorts indicate that approximately 0.8–1.0% of these individuals receive a PC diagnosis within 3 years, corresponding to an approximately six- to eightfold increase in relative risk compared with the general population [79,80]. This excess risk is concentrated within the first 3 years after diabetes onset and is lower among individuals with long-standing diabetes [76,80].

The ENDPAC (Enriching New-Onset Diabetes for Pancreatic Cancer) score was developed using three routinely available variables: age at diabetes onset, change in body weight, and change in blood glucose. In the derivation cohort, a score of ≥3 yielded 80% sensitivity and 80% specificity, with an AUC of 0.87. In an independent validation cohort, the same threshold identified seven of nine PC cases, corresponding to 78% sensitivity and 85% specificity, and increased the observed 3-year PC prevalence from 0.82% in the overall cohort to 3.6% in the high-score group [79]. Preliminary findings from PANDOME do not establish ENDPAC as a stand-alone trigger for imaging: among 109 participants with new-onset or deteriorating diabetes, one stage I PDAC was detected in the deteriorating-diabetes group, whereas no cancer was identified among the 97 participants with new-onset diabetes [11,79,80]. A decision-model analysis estimated that targeting individuals with a predicted 3-year PDAC risk of approximately 1–2% might be cost-effective under specified assumptions, but this does not constitute evidence of clinical effectiveness [81].

The PRIME model (PDAC Risk Model for Earlier Detection) was developed and validated using routinely collected data from approximately 11 million adults across 54 US health systems and the UK Biobank. It incorporates 19 clinical and laboratory variables and achieved a 36-month AUC of 0.75 in the US validation cohort. Individuals in the highest 1% of predicted risk had a substantially elevated risk of PDAC (HR 7.63, 95% CI 6.85–8.49) compared with the middle-risk reference group [82]. Its proposed role as an entry point to a multistage diagnostic pathway remains to be evaluated prospectively. Table 2 summarizes what each candidate tool has been evaluated for and what remains unestablished.

For any of these tools to become clinically useful, discrimination alone is insufficient. Calibration in the target population, an adequate positive predictive value, decision-curve analysis, quantification of false-positive and incidental findings, and demonstration of an effect on stage at diagnosis and mortality would be required. PC screening is not currently recommended in asymptomatic average-risk adults solely on the basis of new-onset diabetes [78], and MetS does not itself constitute an indication for pancreatic imaging or biomarker testing. Formal surveillance remains reserved for individuals with established hereditary or familial risk.

## 6. Metabolic Status and Body Composition as Prognostic Factors After Diagnosis

### 6.1. Impact on Survival and Disease Progression

Evidence concerning metabolic status and prognosis after PC diagnosis is observational and remains vulnerable to confounding by performance status, comorbidity, and treatment allocation. Reported associations therefore should not be interpreted as evidence that metabolic dysfunction causes poorer outcomes. MetS has been associated with higher tumor stage and lower histological differentiation at diagnosis, and with shorter overall survival after adjustment for standard prognostic variables (HR 1.54, 95% CI 1.09–2.17, *p* = 0.013) [85]. In patients with unresectable PDAC, MetS and distant metastasis remained associated with poorer prognosis after multivariable adjustment [85]. Survival differences between patients with and without MetS were reported within the FOLFIRINOX and gemcitabine–S-1 (GS) groups; however, neither the chemotherapy regimen nor the interaction between MetS and regimen was statistically significant. These findings therefore do not establish that MetS modifies treatment efficacy [85]. Following pancreatectomy, increasing numbers of metabolic components were associated with progressively higher mortality estimates (HR 1.32 for three, 1.64 for four, and 1.96 for five components), while fasting glucose was significantly associated with both overall survival and disease-free survival [86].

The association between diabetes and PC prognosis appears to be time-dependent. A meta-analysis showed that diabetes diagnosed within two years before cancer detection was associated with shorter survival after surgery (RR 1.54, 95% CI 1.24–1.91), whereas longer-standing diabetes was not significantly associated with survival in the pooled analysis [87]. A prospective observational study similarly found that recent-onset diabetes was associated with earlier disease recurrence after resection (HR 1.45, 95% CI 1.06–1.99) [88]. By contrast, a large retrospective study found a smaller association between long-standing diabetes and mortality (HR 1.10, 95% CI 1.03–1.16) [89]. This discrepancy may reflect differences in study population and statistical power: the pooled analysis was restricted to resected patients, whereas the much larger retrospective cohort included a broader clinical population and could detect a smaller association. Among patients with PC and coexisting diabetes, a higher glucose–lipid metabolism index (GLMI) remained associated with poorer prognosis after multivariable adjustment [90].

### 6.2. Influence on Treatment Response and Surgical Outcomes

MetS has been associated with greater perioperative risk following pancreatoduodenectomy, including higher adjusted odds of 30-day mortality and higher rates of serious morbidity and reintubation [91]. A separate database analysis of elective pancreatectomy found associations with surgical-site infection and respiratory failure, while pulmonary embolism was reported in both analyses [91,92]. In a retrospective cohort of patients undergoing laparoscopic pancreatoduodenectomy, MetS was associated with higher unadjusted rates of pancreatic fistula (26.6% vs. 8.4%), abdominal infection (33.3% vs. 10.0%), and 90-day mortality (6.7% vs. 1.2%) [93].

Among patients undergoing pancreatic cancer resection, diabetes mellitus was associated with greater tumor size and higher rates of perineural and lymph-node invasion. Diabetes was also associated with higher unadjusted 30-day mortality (3.2% vs. 0.8%) and shorter unadjusted median survival (18 vs. 34 months) [94]. Differences in tumor characteristics, disease stage, and comorbidity may partly account for the observed survival difference.

Mechanisms by which metabolic dysfunction might reduce the efficacy of systemic therapy have been proposed, although the supporting evidence is predominantly preclinical. In murine models, obesity increased stromal desmoplasia, reduced intratumoral perfusion, and impaired chemotherapy delivery [95]. Obesity-associated visceral adipose tissue has also been shown to release extracellular vesicles that activate mast cells, suppress CD8+ T-cell responses, and promote resistance to immune-checkpoint blockade [58]. These mechanistic findings do not establish that correcting metabolic abnormalities improves treatment response in patients with PDAC.

### 6.3. Cachexia, Obesity Paradox, and Body Composition

Cachexia is a multifactorial syndrome characterized by ongoing loss of skeletal muscle mass, with or without loss of fat mass, and is frequently accompanied by reduced appetite and involuntary weight loss. It is present in approximately 63% of patients at PC diagnosis [96,97]. Cachexia has been associated with earlier chemotherapy failure and higher mortality (HR 2.02, 95% CI 1.17–3.48) compared with the absence of cachexia [98].

Although obesity is generally associated with adverse cancer outcomes, some observational studies have reported longer survival among patients with elevated BMI after PC diagnosis. Higher 5-year overall and cancer-specific survival after pancreatectomy was observed among patients with obesity [99]. In the same cohort, a lower ratio of visceral to subcutaneous fat was associated with more favorable outcomes [99]. This apparent obesity paradox may partly reflect reverse causation, because normal-weight or underweight comparator groups may include patients who had already experienced cancer-related weight loss before surgery. Greater subcutaneous fat may alternatively indicate preserved nutritional status and less advanced cachectic depletion, although this interpretation remains speculative.

Measures of body composition have shown better prognostic performance than BMI alone. Sarcopenia was associated with shorter overall survival (adjusted hazard ratio [aHR] 1.39, 95% CI 1.16–1.66) and progression-free survival (aHR 1.31, 95% CI 1.11–1.55) in PC [100]. Baseline sarcopenia and progressive skeletal-muscle depletion during chemotherapy remained associated with poorer outcomes after accounting for changes in BMI [101]. Observational evidence cannot distinguish whether reduced muscle mass contributes directly to poorer outcomes or primarily reflects tumor burden, systemic inflammation, and nutritional deterioration. In resected early-stage PDAC, sarcopenia was also associated with fewer tumor-infiltrating CD8+ T cells; however, this association does not establish that muscle wasting suppresses antitumor immunity or predicts response to immunotherapy [102].

## 7. Clinical Management and Importance

### 7.1. Metabolic Characterization and Its Limits for PC Risk Stratification

Because MetS comprises heterogeneous metabolic phenotypes, its binary classification has limited value for PC risk stratification. Continuous measures may characterize the severity and cumulative burden of metabolic dysfunction more precisely. The continuous MetS severity score (cMetS-S), an age- and sex-specific instrument, has shown better discrimination than categorical MetS definitions for cardiometabolic events and mortality [83]. However, it has not been validated for predicting PC.

No PC-specific thresholds have been established for cMetS-S or other continuous metabolic measures. Clinical implementation would require prospective external validation, accurate estimation of absolute risk, assessment of calibration and incremental discrimination beyond established risk factors, and evidence that applying a defined threshold produces more benefit than harm. For example, the PRIME model identified a top 1% risk group with a substantially increased relative risk of PDAC (HR 7.63), but relative-risk enrichment alone does not demonstrate sufficiently high absolute risk or net clinical benefit to justify pancreatic imaging [82]. Long-standing metabolic burden must also be distinguished from recent metabolic deterioration, because abrupt hyperglycemia or weight loss may reflect an occult malignancy rather than cumulative etiologic exposure.

MetS should therefore continue to be assessed and treated according to established cardiometabolic indications. Formal pancreatic surveillance remains appropriate only for individuals who meet recognized hereditary or familial eligibility criteria, while atypical new-onset diabetes may warrant clinical vigilance during routine care. Current evidence does not support MetS-based pancreatic surveillance or a specific monitoring strategy, and MetS alone is not an indication for pancreatic imaging or biomarker testing.

### 7.2. Lifestyle Interventions, Metabolic Control, and PC Prevention

Behavioral modification remains the cornerstone of MetS management because of its established cardiometabolic benefits; whether it alters PC incidence has not been tested. In the Diabetes Prevention Program, structured lifestyle intervention reduced the incidence of type 2 diabetes by 58% over approximately three years [103]. The ELM randomized clinical trial subsequently showed that a habit-based lifestyle program achieved MetS remission in 28% of participants at 24 months (OR 1.46, 95% CI 1.01–2.14) [104]. These trials demonstrate that structured behavioral programs can produce sustained metabolic improvement, but neither evaluated cancer outcomes [103,104]. Likewise, the observational association between MetS resolution and lower subsequent PC risk cannot establish that deliberately treating MetS prevents PC [66].

Evidence linking lifestyle factors directly to PC risk is predominantly observational. High adherence to a Mediterranean-type dietary pattern was associated with lower PC risk in a meta-analysis including more than 1.3 million participants (summary risk estimate 0.82, 95% CI 0.76–0.88) [105]. Greater adherence to the Dietary Approaches to Stop Hypertension (DASH) pattern was similarly associated with lower PC incidence in the NIH–AARP cohort (HR 0.85, 95% CI 0.77–0.95) [106]. Higher physical activity was associated with a 9% lower PC incidence in a pooled analysis of 18 prospective cohorts (RR 0.91, 95% CI 0.86–0.97) [107]. These associations remain susceptible to residual confounding by smoking, adiposity, socioeconomic factors, and other health behaviors and do not establish that adopting a particular diet or exercise program reduces PC incidence.

In experimental models, exercise attenuated obesity-associated pancreatic tumorigenesis and stromal inflammation, providing mechanistic plausibility but not evidence of prevention in humans [108]. Among 289,557 UK Biobank participants, a favorable lifestyle was associated with lower PC risk among metabolically healthy individuals but not among participants with MetS [109]. Whether this difference represents genuine effect modification by metabolic status or residual confounding remains uncertain. Lifestyle intervention should therefore be recommended for its established metabolic, cardiovascular, and general health benefits, not specifically for PC prevention.

### 7.3. Pharmacologic Considerations

Several pharmacologic agents used in metabolic management have attracted interest because of possible oncologic effects. However, observational and randomized evidence frequently diverge, and none of these agents is indicated specifically for PC prevention.

In a pooled analysis of 29 observational studies, metformin use among patients with type 2 diabetes was associated with lower odds of PC (OR 0.82, 95% CI 0.69–0.98) [110]. In Kras^G12D transgenic mice, metformin also abolished the tumor-promoting effect of obesity [111]. However, a broader meta-analysis of 166 studies identified substantial heterogeneity and evidence of publication bias [112]. After a median follow-up of 21 years in the Diabetes Prevention Program, neither metformin nor intensive lifestyle intervention reduced overall cancer incidence relative to placebo (HR 0.90, 95% CI 0.73–1.10 and HR 0.96, 95% CI 0.79–1.18, respectively) [113]. A meta-analysis of 27 randomized trials likewise found no reduction in overall cancer incidence with metformin (RR 1.07, 95% CI 0.87–1.31); results were consistent in analyses restricted to trials at low risk of bias, and trial-sequential analysis indicated sufficient information to exclude a clinically relevant effect [114]. These randomized studies assessed overall rather than pancreatic cancer incidence. The American Diabetes Association therefore states that no recommendation can currently be made regarding the anticancer properties of metformin [78].

Glucagon-like peptide-1 receptor agonists (GLP-1 RAs) were associated with lower PC incidence in a target-trial emulation, with estimates varying by comparator (HR 0.42–0.82) [115]. By contrast, a meta-analysis of 48 randomized trials found no statistically significant association with PC (OR 0.84, 95% CI 0.53–1.35) [116]. Because the included trials were not designed primarily to assess cancer outcomes and had limited follow-up, modest harmful or beneficial effects cannot be excluded. Sodium–glucose cotransporter 2 (SGLT2) inhibitors have shown antiproliferative activity in experimental models, but clinical evidence concerning PC remains insufficient [117]. Sulfonylureas and exogenous insulin have also been associated with higher PC risk [110]; these estimates may reflect hyperinsulinemia but remain particularly susceptible to confounding by indication, diabetes duration, and disease severity.

Observational evidence has associated aspirin use with lower PC risk (HR 0.80, 95% CI 0.72–0.88), although between-study heterogeneity was substantial (I^2^ = 69.9%) and the certainty of evidence was rated low [118]. A stronger inverse association was reported among patients with type 2 diabetes in an analysis treating aspirin exposure as time-varying to reduce immortal-time bias (aHR 0.58, 95% CI 0.49–0.69) [119]. A proposed mechanism involves inhibition of cyclooxygenase-1-mediated platelet activation and attenuation of local inflammation [120]. In the ASPREE randomized trial of 19,114 adults aged 70 years or older, low-dose aspirin was not associated with overall cancer incidence over a median of 8.6 years (HR 0.98, 95% CI 0.92–1.05) and was associated with higher cancer-related mortality (HR 1.15, 95% CI 1.03–1.29), an unexpected finding whose interpretation remains debated [121]. PC was not reported separately, so ASPREE does not directly test aspirin for PC prevention. Statins have anti-inflammatory and mevalonate-pathway effects, and observational studies have suggested a dose-dependent inverse association with PC risk [122]; randomized evidence addressing PC specifically is unavailable.

These agents should be prescribed according to their established metabolic, cardiovascular, renal, or antithrombotic indications. PC prevention is not an evidence-based indication for metformin, GLP-1 RAs, SGLT2 inhibitors, insulin-modifying therapies, aspirin, or statins. Observational estimates remain vulnerable to immortal-time, time-window, and time-lag biases, as well as confounding by indication. Several of these biases can make users of established first-line or preventive therapies appear at lower risk than comparison groups and may partly explain why observational signals have not been reproduced in randomized evidence.

### 7.4. Limitations of Current Evidence

Human evidence linking MetS with PC risk and prognosis is predominantly observational and remains vulnerable to confounding, selection bias, and reverse causation. An umbrella review of 21 systematic reviews with meta-analysis evaluated 25 associations between MetS and obesity-related cancer risk and five associations with survival. Egger’s and excess-significance tests were positive for 8 of the 25 risk associations (32%) and 3 of the 5 survival associations (60%) [5]. These proportions apply to obesity-related cancers collectively and should not be interpreted as pancreatic-cancer-specific estimates. The survival result was also based on only five associations. Positive small-study and excess-significance tests are compatible with publication bias but may also reflect genuine heterogeneity or variable methodological quality. No equivalent appraisal restricted to PC is available; consequently, the extent of small-study effects in this literature remains unknown.

The absence of a universally adopted MetS definition limits comparability across studies [123]. Component thresholds, phenotype distributions, and the relative contribution of individual abnormalities vary among populations [8,65]. Outcome ascertainment in several large cohorts relied on administrative or reimbursement codes, preventing reliable restriction to histologically confirmed PDAC. Temporal classification is another major limitation: long-standing metabolic dysfunction, recent deterioration caused by occult malignancy, and metabolic changes following diagnosis represent different clinical settings but are not consistently separated. Prognostic analyses are additionally susceptible to confounding by tumor stage, performance status, treatment allocation, comorbidity, and cancer-related weight loss. A summary of evidence by domain is provided in Table 3.

Most mechanistic evidence derives from in vitro and animal experiments, with limited confirmation in human pancreatic tissue. No randomized controlled trial has evaluated whether correction of MetS reduces PC incidence. Mendelian randomization has not resolved this uncertainty: European analyses reported associations with genetically predicted BMI and fasting insulin [68], whereas a Japanese analysis identified an association with genetic liability to type 2 diabetes but not BMI [70]. Such discordance may reflect ancestry, phenotype definitions, instrument selection, and statistical power. Genetic estimates represent lifelong liability and cannot substitute for prospective trials of metabolic interventions.

## 8. Future Research Directions

### 8.1. Targeting Insulin/IGF-1 Signaling

The insulin/IGF-1 axis has been implicated as a biologically plausible link between metabolic dysfunction and pancreatic carcinogenesis. Increased IGF-1 receptor (IGF-1R) expression and activation have been reported in PC, with downstream PI3K/AKT and MAPK signaling promoting cellular survival and proliferation [17,18,19]. However, much of this evidence is derived from tumor samples, cell lines, and animal models and does not establish that IGF-1R signaling is a clinically actionable dependency in human PDAC.

Several IGF-1R-directed strategies have shown substantial activity in preclinical models. Ganitumab (AMG 479), a fully human anti-IGF-1R monoclonal antibody, reduced pancreatic xenograft volume by approximately 80% as monotherapy, with additional activity when combined with gemcitabine [126]. BMS-754807, a dual IGF-1R/insulin-receptor inhibitor, reduced the gemcitabine IC_50_ by more than 100-fold in PC cell lines, while combined treatment achieved 94% net tumor-growth suppression in a murine pancreatic-cancer xenograft model [127].

Ganitumab, however, did not improve survival when added to gemcitabine in a phase III trial in metastatic pancreatic cancer, which was discontinued at an interim analysis for futility [128]. This failure illustrates the limited translation of substantial preclinical IGF-1R inhibition into clinical benefit. Potential explanations include compensatory activation of alternative signaling pathways and the absence of validated biomarkers identifying IGF-1R-dependent tumors. Dual inhibition of IGF-1R and ErbB3 has subsequently enhanced the activity of gemcitabine and nab-paclitaxel in preclinical PC models, but this strategy has not been clinically validated [129].

Research priorities include identifying tumors that remain dependent on insulin/IGF-1 signaling, prospectively validating response biomarkers, and evaluating rational combination strategies. IGF-1R-directed combinations remain investigational, and the available evidence does not support their use for PC prevention or treatment outside clinical trials.

### 8.2. Modulating Adipokines

Obesity-related increases in leptin and reductions in adiponectin have been associated with a tumor-promoting microenvironment, although most pancreas-specific evidence remains preclinical [29,30]. The adiponectin-receptor agonist AdipoRon inhibited leptin-induced pSTAT3 signaling and reduced pancreatic tumor growth in vivo [33]. Experimental findings concerning adiponectin have not, however, been uniformly protective; context-dependent pro-survival effects have also been reported in murine models [32]. In experimental PC models, inhibition of leptin-mediated JAK2/STAT3 signaling reduced cellular invasion and matrix metalloproteinase-13-mediated metastatic activity [34]. Resistin has likewise been linked to tumor-cell proliferation and chemoresistance in experimental systems [30].

Evidence summarized in a recent review indicates that weight loss achieved through bariatric surgery or glucagon-like peptide-1 receptor agonists can be accompanied by increased adiponectin and reduced leptin concentrations [130]. Whether these changes influence pancreatic carcinogenesis independently of weight loss and broader metabolic improvement is unknown. No clinical study has demonstrated that altering circulating adipokine concentrations reduces PC incidence.

Timing-dependent effects have been reported in animal models. An omega-3 fatty acid-rich diet introduced before the development of obesity reduced pancreatic acinar-to-ductal metaplasia and shifted adipose tissue toward a more anti-inflammatory macrophage profile. Introducing the dietary intervention after obesity was established did not reverse early pancreatic lesions [131]. These findings generate hypotheses concerning intervention timing but cannot define a preventive window in humans.

Research priorities include validation of adipokine pathways in human pancreatic tissue, identification of biomarkers reflecting pathway activity, and determination of whether adipokine modulation provides benefit beyond established metabolic effects. Adipokine-directed interventions remain preclinical research strategies and should not be used specifically for PC prevention outside clinical studies.

### 8.3. Anti-Inflammatory and Antioxidant Strategies

Chronic low-grade inflammation and oxidative stress provide a mechanistic rationale for investigating anti-inflammatory and antioxidant strategies in the metabolic context of pancreatic carcinogenesis [31,41,59]. NOX-derived reactive oxygen species can activate pancreatic stellate cells and contribute to an oxidative microenvironment that supports pro-tumorigenic signaling. Accordingly, NOX inhibition has been explored in experimental systems, but no clinical evidence currently demonstrates that this approach prevents PC or improves outcomes in patients with established disease [41].

Among anti-inflammatory agents, aspirin has the most extensive randomized cancer-prevention evidence. In ASPREE, however, low-dose aspirin did not reduce overall cancer incidence, and pancreatic cancer was not analyzed separately [121]. No antioxidant strategy has been shown in randomized trials to prevent PC. Dietary polyphenols, including curcumin, resveratrol, and epigallocatechin gallate, have shown antioxidant and anti-inflammatory activity in preclinical PC models through modulation of pathways such as NF-κB, MAPK, and PI3K/AKT [132]. However, heterogeneous formulations, limited bioavailability, and the doses used in experimental models restrict extrapolation to humans.

These findings should therefore be regarded as hypothesis-generating rather than as evidence supporting antioxidant- or polyphenol-based chemoprevention or treatment. Prospective studies would need to establish pharmacologically achievable exposure, target engagement, safety, and clinically relevant efficacy before NOX inhibitors or antioxidant interventions could be considered for PC prevention or therapy in populations with MetS.

### 8.4. MicroRNA-Based and Microbiome-Targeted Approaches

Adipose tissue-derived microRNAs represent potential mediators of the relationship between metabolic dysfunction and cancer. Obesity alters the microRNA secretion profile of adipose tissue and the composition of circulating microRNAs, although these changes are not specific to PC [50]. In an experimental study, cancer-associated adipocytes transferred exosomal miR-199a-3p to pancreatic tumor cells, promoting proliferation and chemoresistance through the SOCS7/STAT3/SAA1 signaling network [52]. This finding identifies a potential intercellular signaling mechanism but does not establish miR-199a-3p as a clinically validated biomarker or therapeutic target. MicroRNA mimics, antagomirs, and nanoparticle-based delivery systems remain under preclinical investigation; progress would require solutions to tissue-specific delivery, stability, toxicity, and off-target effects, which remain unresolved for pancreatic applications [50].

Microbiome-directed strategies face a different obstacle: the target itself is not defined. A meta-analysis of 10 case–control studies found no statistically significant difference in microbial richness or α-diversity between patients with PC and controls, while differences in the relative abundance of individual genera varied across studies and populations [60]. Genetic and multi-method causal-inference analyses have identified candidate relationships among microbial taxa, circulating metabolites, and PC risk, but these remain sensitive to instrument validity, population differences, and the biological interpretation of taxonomic associations [61]. Microbial products, including lipopolysaccharide and trimethylamine N-oxide, may influence tumor-associated inflammation and antitumor immunity, but their effects are context-dependent and have not been uniformly tumor-promoting in preclinical models [63].

Proposed interventions include fecal microbiota transplantation, probiotics, prebiotics, selective antibiotics, dietary modification, and engineered bacterial vectors [133]. An early-phase FMT study is evaluating safety, feasibility, and biological effects in patients with resectable PDAC, but no completed clinical trial has demonstrated that microbiome modification prevents PC or improves oncological outcomes. Interpretation is further complicated because the microbiome is influenced by diet, medications, antibiotic exposure, geography, and host metabolic status, so causal drivers cannot readily be distinguished from consequences of disease or effects of treatment [62,134]. Most human microbiome data in PC were also obtained at or after diagnosis; consequently, the microbial patterns proposed as intervention targets have not been established as antecedents of disease. Longitudinal sampling before diagnosis, standardized analytical methods, and independent validation would be prerequisites for efficacy-oriented interventional studies.

### 8.5. Biomarker Development for Early Detection

Biomarker studies in this field address two distinct intended uses: estimating the future risk of PC and detecting an occult malignancy. In a prospective Chinese cohort, adding a three-metabolite panel comprising the aspartate-to-alanine ratio, androstenediol monosulfate, and glycylvaline improved the AUC of a model for incident PC from 0.573 to 0.721 [135]. In a UK Biobank cohort of individuals with type 2 diabetes, a metabolic risk score based primarily on triglyceride-enriched very-low-density lipoprotein particles and GlycA was associated with long-term PC risk (highest versus lowest tertile: HR 1.79) and increased the 15-year prediction AUC from 72.3% to 75.8% [136]. These models provide risk enrichment rather than evidence that an occult tumor can be detected at the time of testing.

The prospective, multicenter METAPAC phase 4 diagnostic study evaluated two plasma multimetabolite signatures in 1129 participants with pancreatic lesions requiring further diagnostic assessment. The m-Metabolic signature achieved an AUC of 0.846, with 59.9% sensitivity at 93.6% specificity, compared with an AUC of 0.799 for CA 19-9 alone [84]. An exploratory analysis in a separate cohort of 242 individuals with new-onset diabetes suggested discrimination between participants who developed PDAC and those who did not; however, only three incident cases occurred, precluding a reliable estimate of clinical performance [84]. A five-metabolite panel developed in 133 older adults with new-onset diabetes, including 60 participants with PDAC, achieved an AUC of 0.853 in a hold-out validation set but has not undergone external multicenter validation [137].

Other platforms remain at an earlier stage of development. A retrospective multi-omics study of 88 participants identified a 38-analyte serum signature combining proteins, lipids, and metabolites, but its performance requires validation in independent and prospectively sampled populations [138]. A meta-analysis of seven miRNA studies reported a pooled sensitivity of 0.88, specificity of 0.91, and AUC of 0.95 for early PC [139]. However, these estimates were derived from diagnostic-accuracy studies in which cancer status was already established rather than from testing asymptomatic populations before diagnosis and are therefore likely to overstate performance in a prospective early-detection setting. The contributing studies also evaluated heterogeneous miRNA panels and showed substantial between-study heterogeneity [139]. Prediagnostic serum analyses in two prospective cohorts identified 66 metabolites associated with subsequent PDAC [140]. Separately, a combined SNP–metabolite model achieved an AUC of 0.738 but has not undergone prospective clinical validation [141]. The principal biomarker strategies, their intended-use populations, and their main validation limitations are summarized in Table 4.

High discrimination within a selected diagnostic or case–control cohort does not establish suitability for early detection in a low-prevalence population. Future development should begin with a predefined intended-use population and should assess whether a biomarker adds clinically meaningful information beyond validated clinical risk models. External validation must include calibration, positive predictive value, decision-curve analysis, assay reproducibility, and the consequences of false-positive results. Ultimately, prospective pathway studies must demonstrate that biomarker-guided evaluation increases the proportion of cancers diagnosed at a resectable stage or reduces mortality without producing an unacceptable burden of imaging, invasive procedures, and incidental findings.

### 8.6. Priorities for Longitudinal and Interventional Research

Future research should preserve the distinction among long-term etiology, detection of occult disease, and outcomes after diagnosis. For the etiologic question, prospective cohorts require repeated measurements of glycemia, insulin resistance, adiposity, lipid profiles, and medication exposure, with sufficiently long exclusion periods before PC diagnosis to reduce reverse causation. Existing randomized evidence on metformin, GLP-1 receptor agonists, and aspirin derives from studies that were not designed to test PC prevention and therefore cannot establish whether modifying individual components of MetS changes PC incidence [113,114,116,121]. Changes in insulin, adipokines, inflammation, or body weight may support target engagement but cannot substitute for incident PC as the relevant prevention endpoint. Because PC is a relatively infrequent outcome in prevention cohorts, a trial using PC incidence as its primary endpoint would require a very large sample and prolonged follow-up; linkage of cardiometabolic trials to cancer registries and harmonized analyses across trials may therefore provide more feasible evidence.

For early detection, prospective studies should evaluate complete clinical pathways rather than discrimination alone. ENDPAC and PRIME require implementation studies that establish how risk thresholds would trigger biomarker testing or imaging and whether such pathways perform consistently across healthcare systems and demographic groups [79,80,82]. Similarly, the diagnostic performance demonstrated by METAPAC must be followed by evaluation in the intended-use population and by assessment of downstream clinical consequences [84]. Relevant outcomes include calibration, positive predictive value, number needed to image, false-positive investigations, incidental pancreatic findings, stage distribution, resection rate, and disease-specific mortality. Biomarker and clinical models should be assessed both individually and sequentially because acceptable performance at one stage does not establish the value of the complete pathway.

After diagnosis, longitudinal studies should distinguish pre-existing metabolic status from tumor-driven metabolic deterioration. Repeated measurements of skeletal muscle, visceral and subcutaneous adipose tissue, weight, inflammatory markers, and nutritional intake should be obtained at standardized time points before and during treatment. Analyses require adjustment for disease stage, treatment exposure, performance status, systemic inflammation, and cachexia and should account for treatment-dependent and time-varying changes. Interventional studies of nutritional support, resistance exercise, or anti-cachexia strategies should prioritize muscle function, treatment completion, quality of life, and survival rather than weight gain alone.

Mechanistic human studies integrating pancreatic tissue, single-cell transcriptomics, spatial profiling, circulating metabolomics, and carefully characterized metabolic exposures may help determine which experimental pathways are relevant in humans. Integrating genetic data with environmental and lifestyle exposures may also generate hypotheses about interactions relevant to PDAC [142]. Such studies require sampling that is explicitly timed relative to diagnosis and treatment, appropriate non-cancer and disease controls, standardized assays, and independent replication. The central requirement across all three research settings is alignment between the study population, temporal design, and clinical endpoint: prevention studies must assess incident disease, early-detection studies must demonstrate benefit across the diagnostic pathway, and prognostic interventions must improve outcomes after diagnosis.

## 9. Conclusions

MetS is consistently associated with higher PC risk in observational studies, with pooled relative-risk estimates of 1.25–1.34 and an apparent gradient across the number of metabolic abnormalities. However, MetS is a composite classification that can represent different combinations of exposures rather than a single biological entity or intervention target. Heterogeneous definitions, residual confounding, administrative outcome ascertainment, and reverse causation limit causal interpretation. Mendelian-randomization findings are not uniform across populations and reflect lifelong genetic liability rather than the expected effect of metabolic treatment later in life. Sex-stratified analyses have reported stronger relative associations in women than in men, but whether this represents a genuine biological interaction, differences in the distribution of MetS components, or residual confounding remains unresolved. Proposed mechanisms involving insulin signaling, adipokines, inflammation, oxidative stress, epigenetic regulation, and the microbiome provide biological plausibility but remain predominantly preclinical and are not specific to pancreatic carcinogenesis.

Metabolic changes preceding diagnosis represent a separate clinical setting. New-onset diabetes, rising HbA1c, and involuntary weight loss may be manifestations of occult PDAC rather than causes of the disease. Clinical risk models and emerging biomarkers can enrich populations according to relative risk, but their positive predictive value remains constrained by the low absolute incidence of PC. No risk-enrichment or biomarker-guided pathway has yet demonstrated improvement in stage at diagnosis, resection rate, or mortality.

After diagnosis, MetS components and body composition have been associated with survival, treatment tolerance, and postoperative outcomes. Imaging-based measures of skeletal muscle and adipose-tissue distribution have shown better prognostic discrimination than BMI in observational cohorts. Nevertheless, these associations are vulnerable to confounding by disease stage, systemic inflammation, nutritional status, and cancer-related cachexia. It has not been established that modifying metabolic status or body composition improves oncological outcomes.

The current evidence therefore does not support MetS-based PC screening, imaging, biomarker testing, or pharmacologic prevention. Lifestyle and pharmacologic therapies discussed in this review should be used for their established metabolic, cardiovascular, renal, or antithrombotic indications, independently of the oncological associations reviewed here. Clinical vigilance remains appropriate when new-onset diabetes in an older adult is accompanied by atypical features such as continuing weight loss or rapidly worsening glycemic control, but this does not constitute a validated surveillance program.

Future studies must align their design and endpoints with the clinical question being addressed. Etiologic studies should determine whether modifying specific metabolic components changes incident PC risk rather than treating MetS as a single exposure and should investigate the basis of the observed sex-stratified differences. Early-detection studies must evaluate complete clinical pathways and their effects on stage distribution and mortality, not discrimination alone. Prognostic interventions must demonstrate improvements in treatment delivery, function, quality of life, or survival. IGF-1R inhibition, adipokine modulation, antioxidant or microbiome-directed interventions, and multi-omics biomarker panels remain investigational. Until evidence from appropriately designed studies is available, metabolic dysfunction should be regarded as a risk-associated and prognostically informative state, not as an established target for PC prevention or individualized oncological management.

## Figures and Tables

**Table 1:** PC risk associated with MetS and its individual components.

Exposure or Comparison	Effect Estimate (95% CI)	Study Design and Population	Outcome Definition and Main Interpretive Limitation	Reference
A. Composite MetS and cumulative metabolic burden				
MetS versus no MetS	RR 1.34 (1.23–1.46); I^2^ = 38.8%	Meta-analysis of seven cohort studies	PC definitions varied among contributing studies; histology was not consistently reported	[8]
MetS versus no MetS	RR 1.25 (1.20–1.30)	Pancreatic cancer subgroup of a meta-analysis comprising 31 prospective studies across several gastrointestinal cancer sites	The 31 studies were not all pancreatic cancer studies; the estimate represents the pancreatic subgroup	[9]
MetS versus no MetS	HR 1.37 (1.34–1.39)	Retrospective administrative cohort; *n* = 2,707,296	PC identified from administrative claims; histology was unspecified. The high number of coded PC outcomes warrants consideration of possible outcome misclassification	[65]
Number of MetS components versus no components	1 component: HR 1.11 (1.09–1.12); 2 components: HR 1.23 (1.20–1.25); 3 components: HR 1.42 (1.39–1.45); 4 components: HR 1.66 (1.61–1.72); 5 components: HR 2.03 (1.90–2.17)	Same retrospective administrative cohort; *n* = 2,707,296	Apparent dose–response association based on claims-defined metabolic components and PC; residual confounding and outcome misclassification remain possible	[65]
B. Metabolic phenotypes and longitudinal changes in MetS status				
Metabolically unhealthy normal weight versus MHNW	HR 1.52 (1.27–1.81)	Population-based Korean cohort; *n* = 347,434	PC identified from health-insurance data; metabolic phenotype was determined at baseline	[10]
Metabolically healthy obesity versus MHNW	No statistically significant association	Same population-based cohort	The absence of statistical significance should not be interpreted as proof of no association	[10]
Metabolically unhealthy obesity versus MHNW	HR 1.34 (1.12–1.61)	Same population-based cohort	PC identified from health-insurance data; metabolic phenotype was determined at baseline	[10]
MetS recovered between two examinations versus MetS-free at both examinations	HR 1.12 (1.04–1.21)	Nationwide longitudinal cohort; *n* = 8,203,492; MetS assessed at two consecutive biennial health examinations	PC identified using ICD-10 and cancer-reimbursement codes; histology was unspecified. Recovery was observed, not assigned through an intervention	[66]
MetS developed between examinations versus MetS-free at both examinations	HR 1.17 (1.09–1.25)	Same nationwide longitudinal cohort	The interval between examinations does not establish when individual metabolic abnormalities developed	[66]
Persistent MetS versus MetS-free at both examinations	HR 1.30 (1.23–1.37)	Same nationwide longitudinal cohort	Persistent classification may represent longer exposure but does not establish a causal effect of MetS	[66]
MetS versus no MetS, stratified by sex	Women: RR 1.64 (1.41–1.90); men: RR 1.26 (1.03–1.54)	Sex-stratified meta-analysis of cohort studies	Differences between subgroup estimates do not, by themselves, demonstrate a statistically significant sex interaction	[8]
C. Individual metabolic components				
Hyperglycemia	RR 1.55 (1.42–1.70)	Component-specific meta-analysis of cohort studies	Diabetes duration and proximity to PC diagnosis varied; reverse causation is particularly relevant for recently developed hyperglycemia	[8]
Reduced HDL cholesterol	RR 1.24 (1.11–1.38)	Component-specific meta-analysis of cohort studies	Definitions and covariate adjustment differed among contributing studies.	[8]
Hypertension	RR 1.10 (1.01–1.19)	Component-specific meta-analysis of cohort studies	Small association susceptible to residual confounding	[8]
Obesity	RR 1.13 (0.96–1.32)	Component-specific meta-analysis of cohort studies	Confidence interval included the null; obesity definitions varied among studies	[8]
Hypertriglyceridemia	RR 0.96 (0.87–1.07)	Component-specific meta-analysis of cohort studies	No statistically significant association was detected in the pooled analysis	[8]
Genetically predicted BMI	OR 1.34 (1.09–1.65)	Two-sample Mendelian randomization using PanScan and PanC4 data	Outcome defined as exocrine pancreatic adenocarcinoma. The estimate reflects lifelong genetic liability to higher BMI and should not be interpreted as the expected effect of weight reduction later in life	[68]
Genetically predicted BMI-adjusted waist-to-hip ratio	OR 1.00095 (1.00011–1.0018)	Bi-directional Mendelian randomization	PC identified using ICD-10 or self-reported PC diagnosis; histology was unspecified. MR identified effect is only nominally significant	[69]
Genetically predicted type 2 diabetes and BMI, Japanese population	T2D: OR 1.16 (1.08–1.25); BMI: OR 1.00 (0.95–1.04)	Two-sample multivariable Mendelian randomization	Ancestry-specific instruments; genetic liability does not represent the effect of treatment later in life	[70]

Abbreviations: MR, Mendelian randomization; MetS, metabolic syndrome; HR, hazard ratio; RR, relative risk; OR, odds ratio; CI, confidence interval; BMI, body mass index; HDL, high-density lipoprotein; MHNW, metabolically healthy normal weight.

**Table 2:** Risk-stratification and early-detection tools evaluated in metabolic syndrome and new-onset diabetes populations: current validation status and unresolved requirements.

Tool	Evidence Available	Not Established	Reference
cMetS-S (continuous MetS severity score)	Association with cardiometabolic events and mortality; age- and sex-specific	Discrimination or calibration for PC	[83]
Metabolic phenotyping (MUH vs. MHO)	Population-level association between metabolically unhealthy phenotypes and PC risk	Use as an individual triage tool; no operational threshold; PPV not reported	[10]
ENDPAC ≥ 3	Derivation AUC 0.87; validation sensitivity 78%, specificity 85%, and PC prevalence of 3.6% in the high-score group	Prospective use as a stand-alone trigger for imaging and effect on stage at diagnosis or mortality	[79]
PRIME model	US validation AUC 0.75 at 36 months; marked risk enrichment in the highest predicted-risk percentile	Prospective EHR-guided case finding, actionable threshold, and clinical impact	[82]
m-Metabolic plasma signature;	Diagnostic discrimination in patients with pancreatic lesions or suspected PDAC; AUC 0.846 with 93.6% specificity	Performance after clinical risk enrichment and prospective use in asymptomatic populations	[84]

Abbreviations: AUC, area under the receiver operating characteristic curve; cMetS-S, continuous metabolic syndrome severity score; EHR, electronic health record; ENDPAC, Enriching New-Onset Diabetes for Pancreatic Cancer; MetS, metabolic syndrome; PC, pancreatic cancer; PDAC, pancreatic ductal adenocarcinoma. These tools were developed in different populations, with different sampling designs and outcome horizons; their combined performance has not been evaluated and they do not constitute a sequential pathway.

**Table 3:** Summary of evidence by domain: level of evidence supporting the main conclusions of this review.

Domain	Conclusion	Level of Evidence and Main Limitations	References
Biological mechanisms	Insulin/IGF-1 signaling, adipokine dysregulation, oxidative stress, epigenetic alterations, and gut dysbiosis provide biological plausibility for links between metabolic dysfunction and carcinogenesis	Predominantly preclinical evidence from cell lines and animal models; mechanisms are not specific to the pancreas and do not establish causality in humans	[17,18,19,20,21,22,23,24,25,26,27,29,30,31,32,33,34,35,36,37,38,39,40,41,42,43,44,45,46,47,48,49,50,51,52,53,54,55,56,57,58,59,60,61,62,63,64,124,125]
Prognosis: MetS and metabolic components	MetS and diabetes have been associated with survival and perioperative outcomes after PC diagnosis	Observational, frequently multivariable-adjusted evidence; residual confounding by stage, comorbidity, and treatment allocation remains likely	[85,86,87,88,89,90,91,92,93,94,95]
Prognosis: cachexia and body composition	Cachexia and sarcopenia have been associated with poorer outcomes, while body-composition measures have shown better prognostic performance than BMI alone	Observational evidence; potential reverse causation, cachexia-related bias, and variation in body-composition definitions	[96,97,98,99,100,101,102]
Pharmacologic agents	Observational studies have reported inverse associations for metformin, GLP-1 RAs, aspirin, and statins, but these signals have not been confirmed by available randomized evidence	Mixed observational and randomized evidence; trials generally assessed overall cancer outcomes or had limited follow-up rather than directly testing PC prevention	[110,111,112,113,114,115,116,117,118,119,120,121,122]

Abbreviations: BMI, body mass index; GLP-1 RA, glucagon-like peptide-1 receptor agonist; IGF-1, insulin-like growth factor 1; MetS, metabolic syndrome; PC, pancreatic cancer. These categories are descriptive and do not constitute formal GRADE certainty ratings. Preclinical evidence indicates biological plausibility but does not establish causality in humans; observational associations remain susceptible to residual confounding and reverse causation.

**Table 4:** Emerging biomarker strategies for early detection of PC in MetS and new-onset diabetes populations.

Biomarker Strategy	Intended Setting and Population	Key Result	Current Limitation	Reference
Three-metabolite model	Long-term risk prediction in a prospective Chinese cohort	AUC increased from 0.573 to 0.721	Discovery model; external validation and clinical utility not established	[135]
Metabolic risk score based on 30 metabolites	Long-term risk prediction among UK Biobank participants with type 2 diabetes	HR 1.79 for highest versus lowest tertile; 15-year AUC increased from 72.3% to 75.8%	Developed in a single cohort; incremental improvement in discrimination was modest	[136]
m-Metabolic signature	Diagnostic discrimination in a multicenter population with pancreatic lesions requiring further assessment	AUC 0.846; sensitivity 59.9% at specificity 93.6%; CA 19-9 alone AUC 0.799	Evaluated in an enriched diagnostic population; performance in population-based testing and clinical impact not established	[84]
m-Metabolic signature without CA 19-9	Exploratory analysis in a new-onset diabetes cohort (*n* = 242)	Suggested discrimination between participants with and without incident PDAC	Only three incident PDAC cases; clinical performance cannot be estimated reliably	[84]
Five-metabolite machine-learning panel	Older adults with new-onset diabetes (*n* = 133)	AUC 0.853 in a hold-out validation set	Single-center development with internal rather than external validation	[137]
Multi-omics 38-analyte signature	Retrospective serum comparison of participants with and without PDAC (*n* = 88)	Combined protein, lipid, and metabolite signature	Small retrospective discovery study; prediagnostic performance unknown	[138]
Circulating miRNA biomarkers	Meta-analysis of seven studies evaluating early PC	Sensitivity 0.88; specificity 0.91; AUC 0.95	Known disease status in contributing diagnostic studies, heterogeneous miRNA panels, and substantial between-study heterogeneity	[139]
Prediagnostic serum metabolomics	Two prospective cohorts	Sixty-six metabolites associated with subsequent PDAC; combined models outperformed risk factors alone	Association and model-development evidence; prospective clinical validation required	[140]
Combined SNP–metabolite model	Genomic and metabolomic risk assessment	AUC 0.738	Discovery-stage model without demonstrated prospective clinical utility	[141]

Abbreviations: AUC, area under the receiver operating characteristic curve; CA 19-9, carbohydrate antigen 19-9; HR, hazard ratio; PC, pancreatic cancer; PDAC, pancreatic ductal adenocarcinoma; SNP, single-nucleotide polymorphism.

## Data Availability

No new data were created or analyzed in this study. Data sharing is not applicable to this article.

## Abbreviations

The following abbreviations are used in this manuscript:

ADA	American Diabetes Association
AGEs	Advanced Glycation End Products
aHR	Adjusted Hazard Ratio
AKT	Protein Kinase B
AMPK	AMP-Activated Protein Kinase
ATM	Ataxia Telangiectasia Mutated
AUC	Area Under the Curve
BER	Base Excision Repair
BMI	Body Mass Index
BRCA1/2	Breast Cancer Gene ½
CA 19-9	Carbohydrate Antigen 19-9
CD8+	Cluster of Differentiation 8 Positive
CDKN2A	Cyclin-Dependent Kinase Inhibitor 2A
CI	Confidence Interval
cMetS-S	Continuous Metabolic Syndrome Severity Score
COX-1	Cyclooxygenase-1
ctDNA	Circulating Tumor DNA
DASH	Dietary Approaches to Stop Hypertension
DNA	Deoxyribonucleic Acid
dNTP	Deoxyribonucleotide Triphosphates
EDRN	Early Detection Research Network
EHR	Electronic Health Records
ENDPAC	Enriching New-Onset Diabetes for Pancreatic Cancer
ErbB3	Erb-B2 Receptor Tyrosine Kinase 3
ERK1/2	Extracellular Signal-Regulated Kinase ½
EUS	Endoscopic Ultrasound
EVs	Extracellular Vesicles
FMT	Fecal Microbiota Transplantation
FOLFIRINOX	Folinic Acid, Fluorouracil, Irinotecan, Oxaliplatin
GLP-1	Glucagon-Like Peptide-1
GLMI	Glucose-Lipid Metabolism Index
GlycA	Glycoprotein Acetyls
GSK3β	Glycogen Synthase Kinase 3 Beta
GWAS	Genome-Wide Association Study
H3K27me3	Trimethylation of Histone H3 Lysine 27
HbA1c	Glycated Hemoglobin
HDL	High-Density Lipoprotein
HDL-C	High-Density Lipoprotein Cholesterol
HR	Hazard Ratio
IC_50_	Half-Maximal Inhibitory Concentration
IGF-1	Insulin-like Growth Factor 1
IGF-1R	Insulin-like Growth Factor 1 Receptor
IL-1β	Interleukin-1 Beta
IL-6	Interleukin-6
IR	Insulin Receptor
IR-A	Insulin Receptor Isoform A
IR-B	Insulin Receptor Isoform B
JAK2	Janus Kinase 2
JNK1/2	c-Jun N-terminal Kinase ½
KRAS	Kirsten Rat Sarcoma Viral Oncogene Homolog
LPS	Lipopolysaccharide
MAPK	Mitogen-Activated Protein Kinase
MCP-1	Monocyte Chemoattractant Protein-1
MetS	Metabolic Syndrome
MHO	Metabolically Healthy Obesity
miRNA	MicroRNA
ML	Machine Learning
MMP-13	Matrix Metalloproteinase-13
MR	Mendelian Randomization
MRI	Magnetic Resonance Imaging
mtDNA	Mitochondrial DNA
mTOR	Mechanistic Target of Rapamycin
MUH	Metabolically Unhealthy
ND4	NADH Dehydrogenase Subunit 4
ND6	NADH Dehydrogenase Subunit 6
NF-κB	Nuclear Factor Kappa-light-chain-enhancer of Activated B Cells
NIH-AARP	National Institutes of Health–American Association of Retired Persons
NOX	NADPH Oxidase
NS	Not Statistically Significant
O-GlcNAcylation	O-linked β-N-Acetylglucosamine Modification
OR	Odds Ratio
PALB2	Partner and Localizer of BRCA2
PANDOME	Pancreatic Cancer Detection in New-Onset Diabetes Mellitus
PanIN	Pancreatic Intraepithelial Neoplasia
PC	Pancreatic Cancer
PDAC	Pancreatic Ductal Adenocarcinoma
PI3K	Phosphatidylinositol 3-Kinase
PPARα	Peroxisome Proliferator-Activated Receptor Alpha
PRIME	PDAC Risk Model for Earlier Detection
pSTAT3	Phosphorylated Signal Transducer and Activator of Transcription 3
RAGE	Receptor for Advanced Glycation End Products
RCT	Randomized Controlled Trial
ROS	Reactive Oxygen Species
RR	Relative Risk
SAA1	Serum Amyloid A1
SGLT2	Sodium-Glucose Cotransporter 2
SNP	Single Nucleotide Polymorphism
SOCS7	Suppressor of Cytokine Signaling 7
STAT3	Signal Transducer and Activator of Transcription 3
STK11	Serine/Threonine Kinase 11
TG	Triglyceride
TMAO	Trimethylamine N-Oxide
TME	Tumor Microenvironment
TNF-α	Tumor Necrosis Factor Alpha
VAT	Visceral Adipose Tissue
VEGF	Vascular Endothelial Growth Factor
VEGFR-2	Vascular Endothelial Growth Factor Receptor 2
VLDL	Very Low-Density Lipoprotein
